# Pre-Workout-Induced Pancreatitis

**DOI:** 10.7759/cureus.44609

**Published:** 2023-09-03

**Authors:** Mohammad Ridha, Gilberto Rivera Gonzalez, Makeswaran Seenivasagam

**Affiliations:** 1 Internal Medicine, HCA Las Palmas Del Sol, El Paso, USA

**Keywords:** pancreas imaging, nutritional supplements, sports activities, physical fitness, adult gastroenterology, amino acid, pre-workout supplement, high intensity workout, exercise training, acute pancreatitis

## Abstract

The use of dietary supplements, including pre-workout formulations, has gained widespread popularity among individuals engaged in sports and fitness. This case report presents a unique instance of pre-workout-induced pancreatitis in a previously healthy young adult. The patient, a 35-year-old male, presented to the emergency department with abdominal pain, elevated pancreatic enzymes, and characteristic radiological findings indicative of acute pancreatitis. The patient's history revealed no prior predisposing factors for pancreatitis such as alcohol consumption or gallstone disease. Extensive diagnostic evaluation excluded other potential causes leading to the suspicion of his pre-workout supplement as the source. Pre-workout supplements contain a blend of stimulants, amino acids, and other metabolic ingredients designed to enhance exercise and muscle performance. Research shows that some of these ingredients, such as amino acids, induce metabolic chain reactions which may damage pancreatic cells. However, there is extremely limited literature regarding these amino acids in combination such as in workout supplements. This case prompts an examination of the potential adverse effects of pre-workout supplements, highlighting the need for increased vigilance among healthcare providers and consumers alike. As the use of these products grows, further research is warranted to allow for safe commercial distribution and to protect consumers from serious harm.

## Introduction

Acute pancreatitis is a condition characterized by inflammation of the pancreas, resulting in abdominal pain, elevated pancreatic enzymes, and potential systemic complications in severe cases. Commonly identified causes of pancreatitis include gallstones, alcohol abuse, and hypertriglyceridemia. However, approximately 10% to 20% of cases remain labeled as "idiopathic," where no clear underlying cause is evident despite extensive investigations [1]. Notably, the consumption of pre-workout supplements has surged in popularity among individuals seeking enhanced exercise performance. These supplements often contain a combination of stimulants and other ingredients that are not subject to regulation by governing bodies such as the Food and Drug Administration (FDA). This case report focuses on a unique presentation of pancreatitis associated with the excessive use of pre-workout supplements.

## Case presentation

A 35-year-old male with no previous medical history presented to the emergency department with severe epigastric pain, rated 8/10, which began the day before around noon. The pain was characterized as constant, stabbing in nature, and radiating to his back. The patient led a health-conscious lifestyle, avoiding greasy foods, engaging in regular daily exercise, and abstaining from drugs and alcohol. There was no associated fever, nausea, vomiting, or diarrhea. He further denied a history of drug use, prescription medications, exposure to toxins, or chronic comorbidities such as vasculitis. The patient denied any significant family history, and more specifically, denied any familial history of pancreatitis.

**Figure 1:**
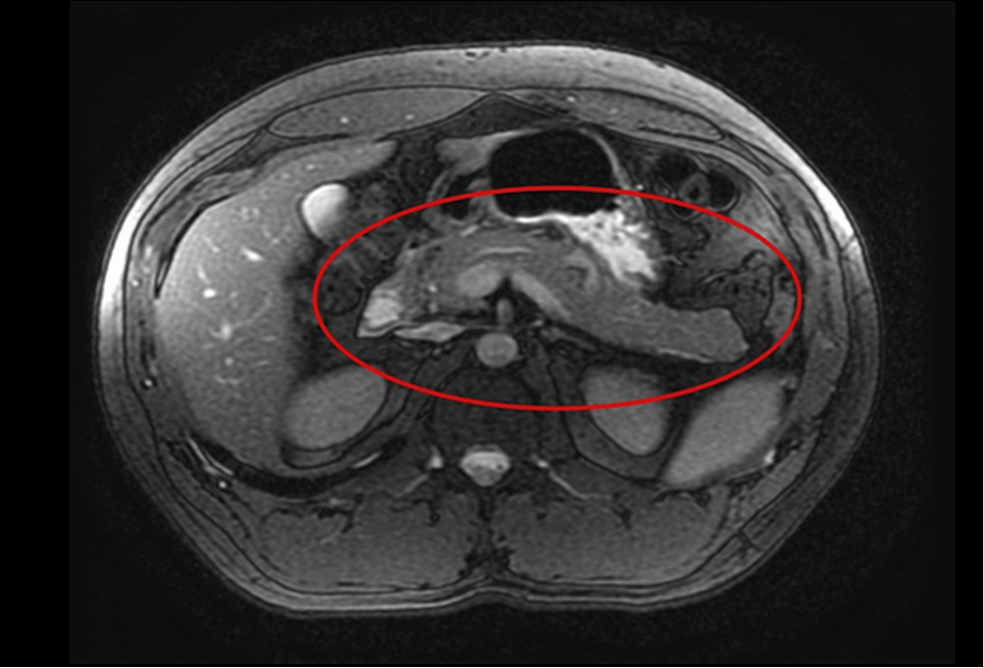
Magnetic resonance cholangiopancreatography Magnetic resonance cholangiopancreatography (MRCP) shows mild peripancreatic fluid suggestive of pancreatitis within the red circle

Laboratory tests revealed a significantly elevated lipase level exceeding 3000 U/L. An ultrasound of the gallbladder was negative for cholelithiasis, cholecystitis, or cholangitis. A follow-up magnetic resonance cholangiopancreatography (MRCP) visualized peripancreatic fluid, indicating pancreatitis (Figure 1). Notably, the gallbladder appeared normal, and there were no signs of common bile duct or pancreatic duct dilatation. Liver enzymes, lipid profile, and an immunoglobulin G4 (IgG4) serum test were all within normal limits. The patient was treated with intravenous fluid resuscitation and pain control, along with a gradual transition to a low-fat diet. Subsequently, the abdominal pain resolved, and the patient was discharged two days later with instructions to follow up with his primary care physician.

Investigating further, the patient reported he recently began a new exercise supplement to boost his performance and training about one month ago. While the patient denied any use of steroids, he admitted to using pre-workout supplements, creatine, and whey protein. He informed us that he used these supplements as directed on the label and did not overuse or double any servings. He provided the names of these products, and we went on to investigate certain ingredients and their potential link to pancreatitis.

## Discussion

In younger patients presenting with abdominal pain, it is crucial to consider atypical causes of pancreatitis such as viral infections, trauma, congenital abnormalities, as well as many others that have proven to have a significant correlation [1]. Although supplement-induced organ failure remains an area with limited research, it is imperative to evaluate such potential factors when assessing patients. The increasing popularity of pre-workout supplements warrants attention, as many of these products lack regulation by the FDA, leaving consumers exposed to insufficiently researched ingredients and potential unknown risks. Therefore, it is vital for physicians, especially when dealing with young adults, to investigate the possibility of supplement-induced pancreatitis.

Pre-workout, a popular category of exercise supplements, aims to enhance muscle contractility and endurance. These products often contain various ingredients such as L-arginine, beta-alanine, L-lysine, and alpha-ketoglutarate. Although data on these ingredients are limited, various amino acids tested on animals suggest significant causation of acute pancreatitis [2]. L-arginine, for example, has a direct correlation with pancreatic edema due to increased vascular permeability resulting from the conversion of L-arginine to nitric oxide (NO) [3]. A case report linked a 16-year-old to pancreatitis induced by L-arginine to NO conversion and its cascade of vasodilatory effects of edema and pancreatic necrosis. L-arginine also has a direct effect on pancreatic beta cells stimulating excess secretion of insulin [4]. Additionally, L-arginine has been shown to potentiate serum thrombopoietin (TPO), which directly increases the extent of pancreatic acinar cell necrosis [5]. In mice samples, L-arginine induced a significant increase in amylase and pancreatic myeloperoxidase causing cellular aberrations, which were confirmed under histopathological studies [6].

Other amino acids, such as beta-alanine, have been linked to pancreatic beta-cell desensitization, resulting in decreased insulin release [7]. The effects of L-lysine when combined with adenosine diphosphate (ADP) directly inhibit adenosine triphosphate (ATP) synthesis in pancreatic mitochondrial cells in mice. The same study correlated L-lysine with inhibition of mitochondrial respiration and a decreased mitochondrial membrane threshold potential [8]. An important precursor to the TCA cycle and ATP production, alpha-ketoglutarate in combination with other amino acids and enzymes leads to excess pancreatic cell proliferation [9].

Although not well-studied, these ingredients have direct effects on pancreatic cells, and in combination, pose a risk for inflammation and pancreatic necrosis. Pre-workout is not adequately tested, yet gaining popularity in the fitness community. We do not know the extent of damage superimposing the above ingredients may cause. Although we discussed a few examples, many pre-workout products include tens to hundreds of ingredients not well-studied in combination.

## Conclusions

This case report provides evidence linking pancreatitis in a 35-year-old male to supplement use, without the presence of typical risk factors. As healthcare professionals, it is essential to consider less common or emerging etiologies when evaluating patients with pancreatitis. This report sheds light on the potential dangers of pre-workout supplements and their association with atypical pancreatitis. As dietary supplement consumption, including pre-workout formulations, continues to rise among athletes and the general population, it is incumbent upon healthcare professionals to be aware of their potential adverse effects. The combination of ingredients mentioned in this report may potentiate the risk of pancreatitis and possibly other organ damage. Identifying alternative etiologies, such as supplement-induced pancreatitis, should be a routine approach in evaluating younger adults with abdominal pain. Further research is warranted to explore the correlation between pre-workout supplements and pancreatitis, with the goal of establishing the safe distribution and regulation of these products.
